# Effect of Microwaves on the Rapid Curing of Metakaolin- and Aluminum Orthophosphate-Based Geopolymers

**DOI:** 10.3390/ma17020463

**Published:** 2024-01-18

**Authors:** Jasmine Aschoff, Stephan Partschefeld, Jens Schneider, Andrea Osburg

**Affiliations:** Department of Construction Chemistry and Polymer Materials, F. A. Finger-Institute for Building Material Engineering, Bauhaus-Universität Weimar, 99423 Weimar, Germany; stephan.partschefeld@uni-weimar.de (S.P.); jens.schneider@uni-weimar.de (J.S.); andrea.osburg@uni-weimar.de (A.O.)

**Keywords:** geopolymer, microwave treatment, metakaolin, aluminum orthophosphate, sodium silicate solution

## Abstract

This paper deals with the influence of microwaves on the hardening and curing of geopolymer binders synthesized from metakaolin or aluminum orthophosphate with sodium silicate solution as the activator. Pure geopolymer pastes as well as geopolymer mortars were considered. The variable parameters were the modulus of the sodium silicate solutions (molar ratio of SiO_2_ to Na_2_O: 1.5, 2.0 and 2.5) and the Si/Al ratio (3/1 and 2/1). Selected samples were cured in a microwave oven until hardening, so the curing time depended on the mixture. For comparison some samples were cured at ambient temperature. To investigate the influence of microwave radiation on the reaction kinetics, isothermal heat flow calorimetry, ultrasonic velocity measurements and rheological investigations into the variation of curing temperature were used. In addition, the mechanical properties of the cured samples were characterized. The results show that microwave curing only takes a few minutes, so it is the most time-saving method. Key factors influencing the geopolymer reaction under microwave radiation are the raw materials as well as the Si/Al ratio. Metakaolin-based geopolymer binders are more stable than those based on aluminum orthophosphate, especially regarding their salt efflorescence. Microwave radiation is an efficient method to accelerate the geopolymer reaction.

## 1. Introduction

Alternative binder systems are being developed worldwide to reduce the large CO_2_ emissions resulting from cement production and to reduce the CO_2_ footprint of the construction industry. Such binder systems are alkali-activated binders, where mineral residual materials and feedstocks like fly ash, ground granulated blast furnace slag (GGBS), natural pozzolana and natural calcined pozzolana (calcined clays) are used and can react with alkaline activators such as alkali silicate solutions, sodium or potassium hydroxides or calcium hydroxide [1,2]. A special type of alkali-activated binders are geopolymer binders, which are classically characterized by a low content of calcium (less than 10 wt.%) [3]. Geopolymers consist of an inorganic, X-ray amorphous three-dimensional aluminum silicate network and are two-component systems [4]. The benefit of the use of industrial side products is that there is no extra manufacturing. For calcined clays, the advantage is their calcination temperature, which ranges from 500 to 800 °C. Therefore, it is much lower than the temperature for cement production [1,5]. These materials are usually activated in an alkaline environment. Another way to synthesize geopolymers is through acid activation, for example, with phosphoric acid [1,6,7]. If geopolymer binders are prepared with alkali silicate solutions, it is also possible to use a solid that contains only aluminum species. Therefore, a berlinite-type aluminum orthophosphate can be utilized [8]. In this paper, synthesis based on both metakaolin and aluminum orthophosphate with activation by three different sodium silicate solutions is considered.

To accelerate concrete hardening in the manufacturing of prefabricated components, a heat treatment is widely used, with the aim of achieving high early strengths quickly [9]. Through higher temperatures the hydration reaction occurs faster and the concrete is set within some hours instead of days [10]. For this thermal treatment, enormous amounts of energy are needed. To reduce the input of energy by focusing on the materials, a microwave treatment can be used [11]. Thermal and microwave heating can also be used for hardening geopolymer binders. Because geopolymer curing occurs through a polycondensation reaction, the thermal treatment contributes to the faster evaporation of water. In contrast, for ordinary concrete the curing reaction is a hydration one, meaning water is needed. So, thermal treatment might be a negative for ordinary concrete, but a positive for geopolymers. Only a few reports describe the working mechanism of microwaves in these materials [12]. Thus, this paper seeks to contribute to understanding these processes.

Microwaves are electromagnetic waves with a wavelength from 1 mm to 1 m, which equals a frequency between 300 MHz and 300 GHz [13]. Through heat’s contribution the movement of atoms and molecules in the geopolymer mixture is accelerated and an effective collision becomes more likely. With the use of microwaves, poorer heat loss compared to conventional heating can be achieved because there is no heat transfer from one object (oven) to another (sample). The heating process of the material occurs faster and more effectively through energy conversion from electromagnetic energy to thermal energy [14,15,16,17].

The heat input in microwave heating is volumetric and does not only arrive from one direction. Materials do not warm from the bottom up, as with the use of heating plates, so there are no large thermal gradients and associated stresses in the sample. Microwaves warm the geopolymers uniformly and volumetrically. This leads to faster solidification of the geopolymer and minimizes the risk of damage [12,18,19,20].

Microwave heating occurs because polar molecules, like water, adsorb microwave radiation and convert it into thermal energy. In the literature, there are three mechanisms mentioned for heating materials through microwave irradiation [17]. The first one is dipolar polarization. Dipole molecules or other polarized compounds that can absorb radiation are set in rotation by a rapidly changing external electric field. There is a continuous change in the polarity or the arrangement of the dipoles because of the constant movement. The movement of the particles causes friction, which leads to the heating of the material. Water is known as the usual substance for this phenomenon, but other polar or ionic substances can also absorb microwave radiation. These include, for example, salts and their solutions, as well as acids and alkalis. Since the latter can also be used as activators for geopolymers, they should be highlighted here [12,17,21,22,23,24]. Second, ionic conduction, which mainly takes place in high-frequency electric fields, must be mentioned. The applied field induces the ions to oscillate, which generates heat. That leads to a warming of the material [12,17,25,26]. The last mechanism is interactive polarization, which is a combination of the two mechanisms mentioned above. As a result of the movement of charged ions, positive and negative charges are formed in a material. Thus, the charge distribution in the field continuously changes. This kind of heating usually occurs when a conductive material is mixed with a nonconductive material [12,17,24,27].

Once the material is warmed up, the progressing reactions are influenced by microwave radiation. Because there are many different raw materials and activators with different water contents for the synthesis of geopolymers, the dielectric properties of geopolymer mixtures vary wildly. Thus, it is difficult to explain the working mechanism of microwaves in real binder systems, because the raw materials and water content seem to be key parameters [12].

**Figure 1:**
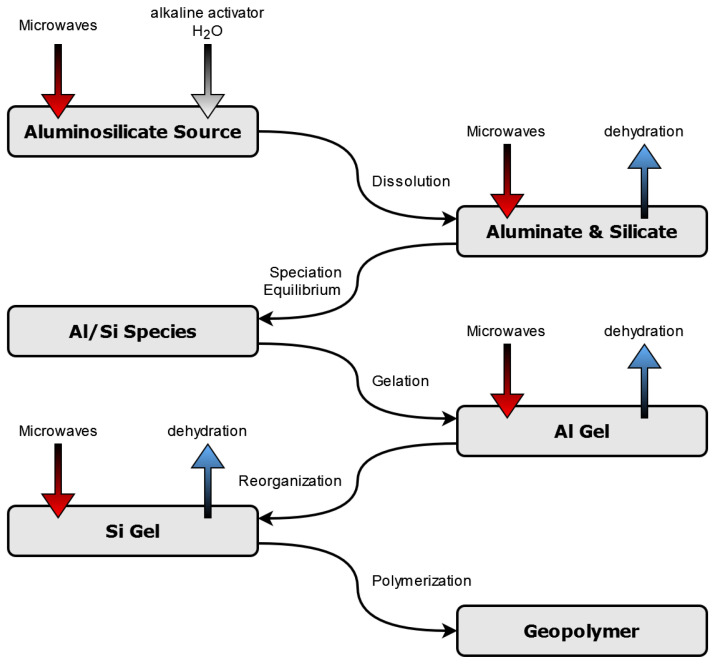
Influence of microwave irradiation on geopolymer synthesis [12,28].

The building mechanism of geopolymers is called geopolymerization. It is composed of different steps, as can be seen in Figure 1 [28]. When the activator is added, the solid binding material is dissolved, and aluminate and silicate monomers are obtained. Water molecules from the activator solution are induced to oscillate by microwave irradiation. Thus, frictional heat is generated, which accelerates the dissolution reaction of aluminate and silicate from the solids. When the solution reaction is finished, the aluminate and silicate species condense into polymers, building the geopolymer network. At elevated temperatures, caused by microwave heating, water evaporates faster and the geopolymer framework grows quickly. This leads to a more dense and homogeneous structure and increases the compressive strength of the geopolymer binders [12,18,29].

In addition, microwave power and irradiation time are important factors that influence the properties of hardened geopolymers. With a longer time and more power, the compressive strength increases [12,30]. However, there exists an optimum, depending on the geopolymer mixture. Above this optimum, the strength decreases as a result of fast water evaporation, and the framework cannot build well [12,19,31]. If the microwave power is constant but the irradiation time increases, first a rise in compressive strength can be observed. The reason for this is the enhanced polycondensation reaction [32,33]. When a certain maximum time is exceeded, a strength reduction is detected. This is because water evaporates too fast and is no longer available for transport processes during the polymerization process. Also, there is volumetric shrinkage due to dehydration, leading to cracking [30,33,34]. If the irradiation time is too short, the solution and geopolymerization reactions are not sufficiently supported, so the hardening process is not accelerated [35]. In contrast, with increasing microwave power but a constant irradiation time, the compressive strength decreases. The reason is fast water evaporation. After a specific maximum power, microcracks in the geopolymer matrix can be detected due to water loss [12,31].

Few authors have reported on geopolymer heat treatments using microwaves at present. Fly ash or slag are mostly used as the aluminum silicate raw material and sodium hydroxide or sodium silicate solutions are used as activators. Sun et al. synthesized geopolymers from fly ash, lead slag and sodium silicate solutions. The first step was curing the species in an oven for 24 h at 75 °C and then using a microwave treatment, reaching a maximum compressive strength of 18.8 MPa with a microwave power of 300 W for 15 min [36]. Hong et al. also used an oven pretreatment before microwave hardening. The raw materials were coal bottom ash and sodium hydroxide solution. After 24 h in an oven at 75 °C and subjected to microwaves with a power level of 200 W for a few minutes, a compressive strength greater than 65 MPa was achieved [37]. Another synthesis of geopolymers with the help of microwave curing was reported by UI Haq et al. In this paper, there was no oven curing before microwaving. The bottom ash of coal-fired power plants was activated by sodium silicate and sodium hydroxide to prepare geopolymers. After this, in five minutes of microwave irradiation at a power of 900 W, thermal isolating foams were obtained with a strength of 3.55 MPa [38]. Also, without conventional heating, the geopolymer synthesis of Onutai et al. occurred. Their raw materials were fly ash, sodium hydroxide and sodium silicate solutions. With one minute hardening under microwave radiation, porous geopolymer binders were formed [18]. For the preparation of geopolymer mortars by adding sand, microwave curing was also utilized. Somaratna et al. produced geopolymer mortars by activating fly ash with sodium hydroxide and the addition of river sand. The mixture was first cured for 12 h at ambient temperatures and then for a maximum of 120 min in a microwave oven, achieving a greater compressive strength compared to a conventional heat treatment [20]. The same raw materials, additionally with sodium silicate solution, Chindaprasirt et al. used, and they treated the geopolymer mortar mixture with microwaves of a power level of 90 W for three to ten minutes to synthesize stable geopolymers [29].

In this paper, both geopolymer binders and geopolymer mortars are considered. In contrast to previous researchers, there is no conventional initial heat treatment before microwave curing. Samples are irradiated until they are hardened.

## 2. Materials and Methods

### 2.1. Materials

A metakaolin was used in this study as an aluminum silicate source (Metaver O, Newchem GmbH, Baden, Austria). In comparison, aluminum orthophosphate was used as an aluminum source (AlPO_4_, Lithopix P26, Zschimmer & Schwarz Chemie GmbH, Lahnstein, Germany).

The chemical components of these materials were analyzed using an optical emission spectrometer with inductively coupled plasma (ICP-OES Activa-M, Horiba Jobin Yvon GmbH, Kyoto, Japan). For mineralogical components, an X-ray diffraction (XRD) was used (X-ray diffractometer, XRD 3003 TT, Seifert Analytical X-ray, Ahrensburg, Germany) in combination with a Rietveld phase analysis using the program AutoQuan^®^ (XRD-Eigenmann, Karlsruhe, Germany). Furthermore, the particle size distribution was identified via laser granulometry (Laser particle analyzer, LS 230, Coulter, Indianapolis, IN, USA). Using the BET method (BET Analyzer, Coulter SA 3100, Beckman Coulter GmbH, Krefeld, Germany) the specific surface area was calculated. A helium pycnometer (AccuPyc 1330 V2, Micromeritics, Norcross, GA, USA) was used to characterize pure density.

The results for the metakaolin are shown in Table 1. Chemically it is manly composed of silica and alumina oxide, corresponding to a high amount of kaolinite in its mineralogical composition. Another big component is X-ray amorphous phases. The metakaolin has a high surface area of approx. 11.5 m^2^/g. To specify the pozzolanic activity of the metakaolin, a modified Chapelle test was carried out according to the French norm NF P 18-513, Annex A [39]. To be ‘pozzolanic’ the material must convert at least 650 mg of calcium hydroxide per gram of material. For the metakaolin, the result was 1250 mg/g, which indicates very good pozzolanic activity.

Table 2 shows the analysis results of the aluminum orthophosphate. Its main chemical components are alumina and phosphorus oxide. The main mineral is berlinite. With nearly 3 m^2^/g the BET surface area of aluminum orthophosphate is much less than that of metakaolin and its particle charge distribution is wider than that of metakaolin.

Sodium silicate solutions (sodium water glasses) were chosen as the activator solutions. The modulus of the sodium silicate solutions (water glass modulus, molar ratio of SiO_2_ to Na_2_O) was 1.5, 2.0 and 2.5, and the solid content was fixed at 40 wt.%. To obtain sodium silicate solution, sodium hydroxide pellets (≥98%, Carl Roth GmbH + Co. KG, Karlsruhe, Germany) were dissolved in ultrapure water, followed by the addition of silica gel (≥99.4%, 400–220 mesh, Carl Roth GmbH + Co. KG, Karlsruhe, Germany). Since the viscosity of the sodium silicate solutions is an important factor in the workability of geopolymer binders, it was analyzed using a rotational viscometer (Rheotec^®^ Brookefield DV III-ultra, Middleboro, MA, USA), resulting in dynamic viscosities of 70.79, 51.87 and 48.89 mPa∙s for the moduli 1.5, 2.0 and 2.5. It is obviously that the viscosity depends on the alkali content. For sodium silicate solutions, the viscosity usually decreases with increasing alkali content. When a certain limit concentration is exceeded, the viscosity increases again [8].

To prepare the geopolymer mortar, crushed granite sand was added (fraction 0–0.5 mm, Granitwerk Fischer GmbH & Co. KG, Wurzbach, Germany). This was necessary because geopolymer binders made of one reactive solid and an activator without sand showed strong drying shrinkage, resulting in cracks and the deformation of the binders. By adding crushed granite sand these effects can be counteracted. Just as for the other raw materials, the properties of the sand were also analyzed. The results can be seen in Table 3. The sand is mainly composed of silica and alumina oxide and its main minerals are quartz, orthoclase and albite. Its BET surface is in the same region as that of the aluminum orthophosphate.

### 2.2. Sample Preparation and Microwave Irradiation

To prepare the geopolymer binder pastes, metakaolin (M) or aluminum orthophosphate (A) was activated with one sodium silicate solution (WG). Therefore, the ratio of Si/Al was fixed at 2/1 for both pastes and additionally at 3/1 for pastes with aluminum orthophosphate. The samples were labeled with the M or A value of the raw material, the value of the modulus of the sodium silicate solutions (WGM) and the Si/Al ratio. To produce the geopolymer pastes, the solid material was mixed with the required amount of sodium silicate solution until a homogeneous paste was obtained. To produce geopolymer mortars, granite crushed sand (S) was added to the pastes. Its amount was the triple amount of solid raw material. The labels of the mortar samples were the same as those of the pastes, but with the addition of an S. When the mixture was homogeneous, it was poured into cylindrical plastic molds. So, samples with a diameter of about 28 mm and a height of approx. 45 mm were obtained. Five molds of each particular mixture were placed in the microwave (MW) cabin (Ultra X 3506, maximum power level: 1750 W). Samples were irradiated until they were set. That is why the curing time varies; it depends upon the composition of the material (Table 4). In preliminary tests, it was found that continuous microwave irradiation leads to a fast increase in temperature to the boiling point of the pore solution, so an interval circuit was defined, dictating 3 s of microwave pulses followed by 7 s breaks. This corresponds to a power level of 30% of the maximum power (525 W). After hardening with microwave radiation, the samples were stored under laboratory conditions. For comparison, samples were also cured at ambient conditions in the laboratory without thermal treatment. On the label of these samples, the prefix RT is mentioned.

This means that the sample M_1.5_S_2-1 is a geopolymer mortar made of metakaolin, sodium silicate solution with the modulus 1.5 and crushed granite sand. Its Si/Al ratio equals 2/1 and it was cured with the help of a microwave oven. In contrast, the sample M_1.5_2-1_RT contains no sand and was not treated by microwaves.

### 2.3. Dielectric Properties

To characterize the geopolymer pastes, some examples’ dielectric properties were analyzed. The permittivity or dielectric constant represents the ability of a material or its molecules to be polarized by an electric field. The dielectric loss factor describes the loss of energy in the material [12,40]. In general, the higher the loss factor, the higher the absorption of microwave energy. Measurement was performed at the Fricke und Mallah Microwave Technology GmbH for two chosen samples (one metakaolin and one aluminum orthophosphate). Two different frequencies (915 MHz and 2450 MHz) were used and the measurement was performed with fresh mixed geopolymer pastes and again one hour after mixing. Furthermore, 2.45 GHz (2450 MHz) represents the typical frequency of a household microwave oven.

It can be taken from Table 5 that as the time after mixing increases, the dielectric constant and loss factor decrease. With higher frequencies there is a reduction in dielectric properties. Thus, there is a dependence of the dielectric properties on the frequency and time between mixing and measurement. When both geopolymer binders are compared, it can be seen that the fresh geopolymer pastes show dielectric constants and dielectric loss factors in the same range. But one hour after mixing there are great differences. This could probably be due the different water glasses or to the different aluminum sources.

These results are confirmed in a study by Jumrat et al. [40]. The authors found that the composition and the time after mixing strongly influence the dielectric properties of geopolymer mortars. Both the dielectric constant and the dielectric loss factor have their highest values right after mixing and decrease afterwards until they reach their minimum 24 h after mixing. The reason for this is that at the beginning of the geopolymer synthesis a lot of free water and monomers are present. Because water is a polar molecule it easily can be polarized by an electric or electromagnetic field, meaning that water is a good dielectric. With the progressive reaction, the free water and the monomers are converted to bound water and polymers, and the geopolymer begins to solidify. Since the solid material has less polar properties than water, it is less influenced by the electric field [40].

### 2.4. Methods

The rheological investigations of the geopolymer pastes were performed by measuring their dynamic viscosity and shear stress with a rotation viscometer (Rheotec^®^ Brookefield DV III-ultra, Middleboro, MA, USA) with a spindle type SC4-18. For pure sodium silicate solutions, measurement was performed at a constant rotation speed of 60 rpm for 10 min. To measure the geopolymer pastes, the experimental setup was modified. The measuring cell was placed in a water bath that was kept at constant temperature, through a hot plate, at 40 °C, 60 °C and 80 °C. To ensure a uniform temperature, the water was stirred continuously. The spindle rotation speed was fixed at 20 rpm. The measurement stopped automatically when the geopolymer pastes hardened and the spindle was unable to rotate anymore or when the torque reached 100 N∙m. Therefore, the measurement time varies depending on the geopolymer mixture.

To follow the reaction’s progress during the hardening of the geopolymer pastes, isothermal heat flow calorimetry investigations (Calorimeter mc cal^®^/100P, C3 Prozess- und Analysetechnik GmbH, Haar, Germany) took place. Therefore, heat development during geopolymerization was detected. The investigations were attempted at temperatures of 20 °C, 40 °C, 60 °C and 80 °C with a running time of three days. The components of the geopolymers were pre-tempered, rapidly mixed outside the calorimeter, and put back to start measurements.

An ultrasonic transit time measurement (Ultratest IP-8, UltraTest GmbH, Achim, Germany) was conducted to follow structure formation during the geopolymerization reaction of the mortars. For this, the geopolymer mortars were mixed, placed in a special measuring cell with an ultrasonic transmitter on one side and an ultrasonic receiver on the other side. The measurement was performed once at 25 °C and once at 60 °C with a running time of two days, respectively.

The compressive strength of the geopolymer mortars was measured using a universal testing machine (TIRAtest 28100, TIRA GmbH, Schalkau, Germany). Therefore, cylindrical samples with a diameter of nearly 28 mm and a height of approx. 45 mm were used. The samples were ground in the parallel plane, so their height varies slightly. Strength testing was carried out at 2 and 7 days for samples with microwave curing and after 7 and 14 days for reference samples cured at ambient temperature.

To obtain information on the microstructure of hardened geopolymers, a scanning electron microscope (SEM) TM3000 (Tabletop microscope, Hitachi High-Technologies Corporation, Tokyo, Japan) was used. It was connected to an energy-dispersive X-ray spectroscopy (EDS) Bruker EDS (Bruker Corporation, Billerica, MA, USA) to analyze the chemical compositions at particular points of the sample.

## 3. Results and Discussion

### 3.1. Temperature Development under Microwave Treatment

With the help of a nickel-chromium-nickel thermocouple (type K) and a multimeter, the development of the sample temperature under the influence of microwave radiation was followed. For this, geopolymer pastes were used. The pastes were mixed in a plastic container and put in the microwave oven for 5 s. Afterwards, the temperature was quickly measured. The sample was put back into the microwave chamber and irradiated for another 5 s before again measuring the sample temperature. To avoid thermal gradients, the sample was stirred before the temperature was measured. To prevent the geopolymer pastes from cooling during temperature measurement, the container was put in Styrofoam for isolation. The measurement was repeated up to a maximum microwave time of 120 s or until the geopolymer pastes had hardened. After each measurement, the temperature sensor was also rinsed in deionized water to remove the adhering geopolymer residues and thus minimize measurement errors.

In Table 6, the results of the measurements are shown. It was found that for the metakaolin-based geopolymer pastes only the sample M_1.5_2-1 was hardened during the measurement. So, it can be assumed that with the increasing modulus of the sodium silicate solution (meaning its decreasing alkalinity), the time until hardening increases. This is confirmed by the aluminum orthophosphate samples with a Si/Al ratio of 2/1. Here, the irradiation times increase with decreasing alkalinity for all samples. In contrast to that, for the Si/Al ratio of 3/1 there was no sample hardened during the measurement time. This leads to the conclusion that the Si/Al ratio influences the hardening time of geopolymer pastes.

Regarding the start temperatures, it can be seen that for all metakaolin samples the start temperature is around the same level. For both series of aluminum orthophosphate-based geopolymer pastes the start temperature decreased with the increasing modulus of the sodium silicate solution. Their temperatures lie above the start temperature of the metakaolin geopolymers. This is because the reaction of aluminum orthophosphate starts right after adding the activator to the solid and leads to a high release of initial wetting and solution heat. Further investigations confirmed this (compare Section 3.3 and Section 3.4).

For the end temperatures there is a difference compared to the behavior discussed above. The metakaolin-based geopolymer pastes show a rise in end temperatures when the modulus of the sodium silicate solution increases from 1.5 to 2.0. But afterwards, with the further rise of the modulus, there is no significant change in the end temperatures. The same effects were detected for the other samples with a Si/Al ratio of 2/1 based on aluminum orthophosphate. Contrastingly, for the geopolymer pastes with a Si/Al ratio of 3/1, there was no substantial change in their end temperatures with the changing alkalinity of the activator solution. From this it can be concluded that the Si/Al ratio of geopolymers is influencing the temperature development.

Moreover, the influence of the sample weight on temperature development was studied. For this, sample M_1.5_2-1 was selected as an example. It was mixed according to the mass proportions of the components in three different gradations (simple basic mixture (33 g) to triple basic mixture (99 g)). The measurement of the temperature was performed as described above. At the end of all measurements, samples were hardened.

**Figure 2:**
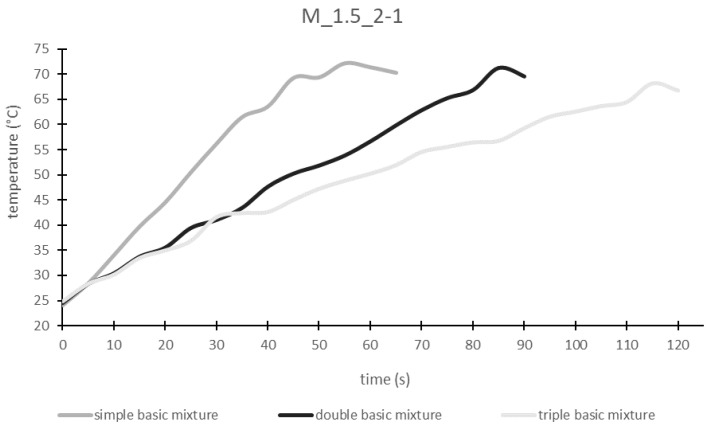
Influence of the sample weight on temperature development.

As can be seen in Figure 2, with increasing the sample weight, the time until hardening increases. This is due to the fact that heating a larger mass requires more time. It can also be seen that the start and end temperatures are in the same range regardless of the mass. They are approx. 24 °C and approx. 70 °C, respectively.

**Figure 3:**
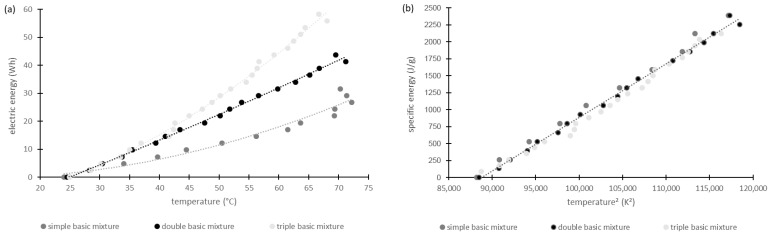
Relationship between (**a**) electric energy, (**b**) specific energy and sample temperature.

From this investigation the relationship between applied energy and sample temperature can be determined. The electrical energy introduced into the sample was calculated (Equation (1)) from the total microwave pulse time and the power of the microwave (1750 W) and plotted as a function of temperature (Figure 3a). There is a quadratic relationship between the energy introduced and the sample temperature. The rise of the parabola increases as the sample weight increases. This means that with a higher sample weight, more energy must be applied to heat it.

(1) $$Electricenergy\left(Wh\right)=\frac{Power\left(W\right)\times Impulsetime\left(s\right)}{3600}$$

If the specific energy, meaning the energy introduced per mass, is plotted against the temperature, the specific heat requirement of the geopolymer paste is obtained as the rise of the regression line (Figure 3b). This value is always the same for a material, regardless of sample weight. For the sample M_1.5_2-1 the specific heat requirement is 0.08 J/(g∙K^2^).

### 3.2. Rheological Behavior

**Figure 4:**
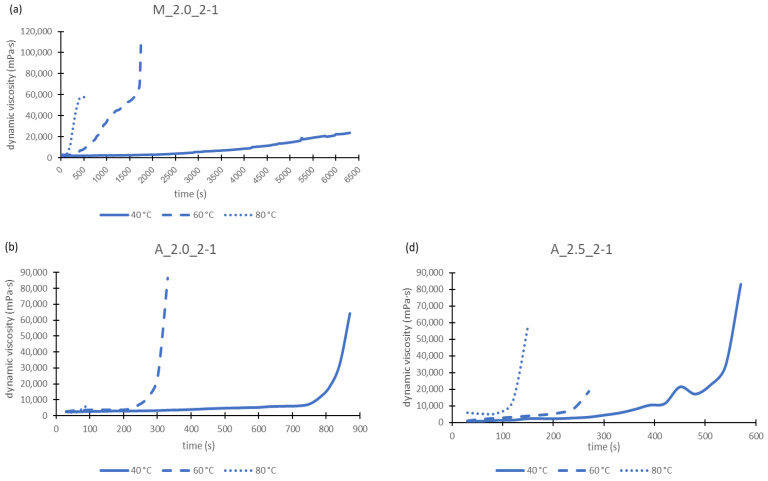

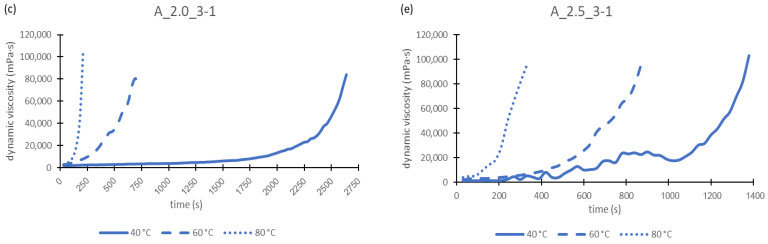
Dynamic viscosities of geopolymer pastes: M_2.0:2-1 (**a**), A_2.0_2-1 (**b**), A_2.0_3-1 (**c**), A_2.5_2-1 (**d**) and A_2.5_3-1 (**e**) in relation to the paste temperature.

For this investigation, all geopolymer pastes were considered, except M_2.5_2-1 as this one reacts very slowly, as could be observed in pretests. The aim was to observe the starting point of structural formation. Measurement took place at 40 °C, 60 °C and 80 °C for each mixture. The geopolymer paste A_1.5_2-1 could not be investigated at 80 °C. Because during the 60 °C measurement the paste had already cured after 90 s, it was assumed that this happens even faster at higher temperatures. Therefore, the 80 °C measurement was omitted for this geopolymer paste, in order not to damage the instrument and to prevent the hardened geopolymer paste from not being removed from the measuring cell. This decision is also confirmed by the fact that with sample A_2.0_2-1 at 80 °C, hardening occurred after just 90 s and the measurement stopped (Figure 4b). With a higher alkalinity of the sodium silicate solution the curing might be accelerated.

Viscosity curves as a function of time show nearly identical courses for all mixtures tested. Therefore, in Figure 4 only exemplary diagrams are shown.

As evident in Figure 4, the time until the geopolymer pastes had hardened decreased with increasing temperature. This was to be expected, since geopolymerization is accelerated by a higher temperature. With a Si/Al ratio of 2/1, the maximum viscosity increases from 40 °C to 60 °C and then decreases again. However, with a ratio of 3/1, an increase in viscosity can also be observed from 60 °C to 80 °C. Consequently, the Si/Al ratio influences the viscosity behavior of the geopolymers.

Considering the different sodium silicate solutions used, for the metakaolin-based geopolymer pastes, with the increasing modulus of the sodium silicate solutions, the time until curing increases. The same observation was made for the aluminum orthophosphate samples with an Si/Al value of 2/1 at elevated temperatures of 80 °C and 60 °C. At 40 °C the results were somewhat different. Here, with increasing the modulus of the sodium silicate solutions, the time first increases (WGM 1.5 to 2.0) and then decreases again (WGM 2.0 to 2.5). This effect was also found for the Si/Al value of 3/1 at 40 °C. For this Si/Al value the results at 60 °C and 80 °C were reversed. This means that the time until curing of the geopolymers first decreases (WGM 1.5 to 2.0) and then rises again (WGM 2.0 to 2.5) with the increasing modulus of the sodium silicate solution. So, the alkalinity of the activator influences the curing of the geopolymers.

Furthermore, the onset points of the geopolymer pastes were determined by applying tangents to the viscosity curves. The onset point marks the start of the formation of the geopolymer network and is the point where the viscosity curves begin to rise continuously. The values in Table 7 should only give direction and cannot be seen as an absolute number, as the error margin of this method can be very high.

In Table 7 one can see that the time decreases with increasing temperature. Therefore, the curing occurs faster. This fact was already mentioned above, but it is now proved for all geopolymer pastes used in this work. The metakaolin-based geopolymers need more time for hardening than the aluminum orthophosphate-based geopolymers at the same Si/Al ratio of 2/1. This fact can also be seen in Figure 4. When aluminum orthophosphate-based pastes with different Si/Al ratios are compared, it is found that a higher Si/Al ratio leads to a slower start of the forming of the geopolymer network. This may be caused by the larger volume of water and the smaller proportion of aluminum orthophosphate contained in the mixtures when the Si/Al ratio is equal to 3/1. Therefore, the geopolymerization reaction is slowed down. This could also explain the lower compressive strengths of these geopolymers (compare Section 3.6).

### 3.3. Isothermal Heat Flow Calorimetry

This investigation was carried out with geopolymer pastes. For better comparability, only the pastes with a Si/Al ratio of 2/1 were used of both the metakaolin and aluminum orthophosphate-based geopolymers. The results of all of samples measured with the same aluminum source were very similar, meaning that the statements made below are valid for all samples investigated. At this point, only selected diagrams of the results are shown, because they all look very similar.

**Figure 5:**
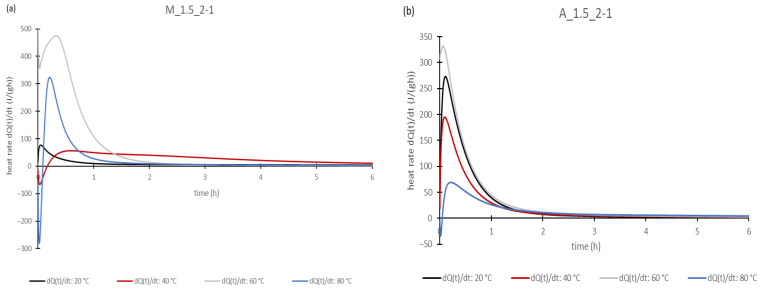
Heat rates of the geopolymer pastes: M_1.5_2-1 (**a**) and A-1.5_2-1 (**b**).

**Figure 6:**
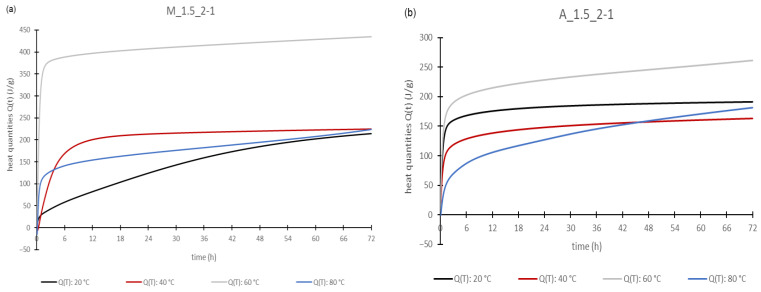
Heat quantities of the geopolymer pastes: M_1.5_2-1 (**a**) and A_1.5_2-1 (**b**).

Figure 5 and Figure 6 represent some of the results of the calorimetric investigations. The obtained curves for the calorimetric investigations show the typical route of those results found in the literature [8,41]. Partschefeld et al. investigated geopolymers based on aluminum orthophosphate activated with sodium silicate solutions with different moduli. The authors found out that with an increasing concentration of aluminum orthophosphate the total heat release rises. It was also found that there was no proportional increase in the degree of conversion by changing the Si/Al ratio from 3 to 2, which indicates an incomplete dissolving of the aluminum orthophosphate. Furthermore, the study compared the different moduli of the sodium silicate solutions, showing that with increasing alkalinity the solubility and the degree of conversation of aluminum orthophosphate increases too [8]. Cai et al. synthesized geopolymers with metakaolin and a mixture of sodium silicate solution and potassium hydroxide as the activator. The authors carried out calorimetric investigations at different temperatures. It was found that the maximum heat peak and cumulative heat increase with rising temperatures. Also, different alkalinities of the activator solution were investigated. With a rising alkali proportion the maximum heat peak and cumulative heat increase [41].

Considering the heating rates in Figure 5, there is a high peak at the beginning of the curves and afterwards the curves flatten until the values are nearly zero after one to two hours. So, Figure 5 only represents the first six hours of measurement. At elevated temperatures at the beginning of measurement, a falling or negative peak can be found. This is due to the mixing procedure occurring outside the tempered measuring chamber, so the samples lost heat and had to be heated up again at the beginning of the measurement. However, at 60 °C, a point seems to be reached where the heat released from the wetting and the solution prevents the sample temperature from dropping too much. The high peaks at the beginning of measurement are due to the strongly exothermic wetting and dissolution reactions that occur when the activator was added to the solids. After the first strong peak, there was a rapid decrease in the heat rate, which is caused by the decrease in the pH of the solution when the aluminate and silicate species are dissolved. This is because for this hydroxide ions are consumed by binding them to the aluminum or silicon species. As the base strength decreases, there are fewer species that can be dissolved [42]. However, heat release continues after the initial phase, albeit with much less intensity, as can be seen from the smaller increase in the curves. This can be explained by the further polycondensation reactions creating an increase in the crosslinking of the geopolymer network.

For all samples, the detected heat quantities (Figure 6) increase with time, so the geo-polymerization reaction is an exothermic reaction. The highest heat output occurred at 60 °C. However, there are some differences between the aluminum orthophosphate- and the metakaolin-based geopolymer pastes. For metakaolin-based pastes (Figure 6a the lowest total heat release was shown at 20 °C, followed by 80 °C and 40 °C. This is in contrast to the results of Cai et al., where a continuous rise in the heat was detected with increasing temperatures [41]. In contrast, geopolymer pastes made with aluminum orthophosphate (Figure 6) showed a higher heat release at 20 °C than at 80 °C and 40 °C. Additionally, the total heat release at the end of the measurement after 72 h was lower compared to the metakaolin geopolymer pastes. But, with regard to the beginning of the reaction, in the first few hours, the heat quantities of the aluminum orthophosphate pastes are higher than those of the metakaolin-based geopolymer pastes (except for 80 °C). This shows that the geopolymerization reaction starts faster with the aluminum orthophosphate- than with metakaolin-based pastes. Heat is emitted faster, right after mixing the fluid and solid components.

To compare the different activators, it was found that the lower the alkalinity of the activator, the slower the reaction proceeds. This was proved by the fact that for both the metakaolin-based and aluminum orthophosphate-based geopolymer pastes, it could be seen that the heat rates decrease with the increasing modulus of the sodium silicate solutions at the same temperature level. This was also found by Partschefeld et al. for aluminum orthophosphate activated with different sodium silicate solutions [8] and by Cai et al. for metakaolin-based geopolymers [41]. The heat quantities increased with increasing the temperature to 60 °C for all samples, before again decreasing at 80 °C. This is probably related to the already-mentioned very large heat losses that occurred during the mixing of the 80 °C samples. It seems to take a long time before this loss can be compensated for by the heat released in the dissolution and wetting reactions and during geopolymerization.

### 3.4. Ultrasonic Transit Time Measurement

For this measurement only the samples M_1.5_S_2-1, A_2.5_S_2-1 and A_2.5_S_3-1 were chosen, because they had the highest compressive strengths after the early age of 2 days. Therefore, it was assumed that the geopolymerization reaction occurs the fastest in them.

**Figure 7:**
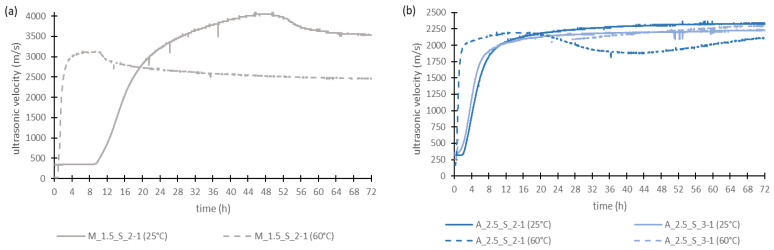
Ultrasonic velocity of the geopolymer mortars based on metakaolin (**a**) and aluminum orthophosphate (**b**).

Figure 7 shows the results of the ultrasonic transit time measurement, where the measurement at 25 °C is shown as solid lines and at 60 °C as dashed lines. When the material begins to solidify, the ultrasonic velocity increases because the formation of the structure and the associated formation of solid phases improve the transmission of ultrasound. As soon as the velocity no longer changes significantly the material is completely hardened.

For all samples, it can be taken from Figure 7 that the ultrasonic velocity first increases very quickly. After a certain time, the rise of the curves flattens. This is because the ultrasonic velocity in liquids is slower than in solids. At the beginning of the measurement, when the geopolymer components are freshly mixed, the water content of the sample is very high. With starting the geopolymerization reaction, solidification began. So, the proportion of solid material increases while the amount of liquid phase decreases. Therefore, the ultrasonic velocity rises strongly at first. After some hours, there is a large amount of material solidified, so the ultrasonic velocity slows down.

Regarding the metakaolin-based geopolymer mortars in Figure 7a, it can be found that at 25 °C it takes approx. nine hours before the ultrasonic velocity rises. With an elevated temperature (60 °C) the rise of ultrasonic velocity starts immediately. A maximum ultrasonic velocity of about 4000 m/s is reached after 50 h at 25 °C. Much earlier, after around 10 h, the maximum (approx. 3100 m/s) for 60 °C is obtained. So, at higher temperatures the curing of the geopolymers is accelerated.

In Figure 7b the two different Si/Al ratios of the aluminum orthophosphate-based mortars are compared. Here, the rise of the ultrasonic velocity starts earlier compared to the metakaolin samples. It can be found that there are no significant differences between the two Si/Al ratios. This indicates that the Si/Al ratio is less important for geopolymer formation. Here, the start of the increase in the ultrasonic velocity for 60 °C is also immediately after measurement starts. So, it is earlier than at 25 °C too, where it takes about 2 h before an increase in the ultrasonic velocity occurs. The maximum value of the ultrasonic velocity is, regardless of temperature, at nearly 2200 m/s; almost at the same level for all samples. Only the time until reaching this value varies. At 25 °C it takes about 32 h, at 60 °C it is after 14 h. This also shows that elevated temperatures lead to an acceleration of the curing of geopolymer binders.

Comparing both the solid raw materials, it was found that the metakaolin-based geopolymer mortar requires a significantly longer time before the setting and curing process begins. Even at the end of the hardening process, when the ultrasonic velocity is nearly constant, clear differences between the two types of geopolymer mortars can be seen. Thus, the setting of the metakaolin geopolymer mortars is much slower than that of the aluminum orthophosphate geopolymer mortars. The higher end value of the metakaolin mortar is a clear sign of a denser structure (see Section 3.7), which corresponds to a higher compressive strength (see Section 3.6). Figure 7 also shows that for all geopolymer mortars the setting and hardening starts significantly earlier at 60 °C than at 25 °C. The end of the hardening reaction is also reached much faster than at 25 °C. The increase in the curves is significantly steeper compared to the tests at 25 °C, suggesting a faster reaction as a result of the increased temperature. This was confirmed in other investigations mentioned above, which had also shown an accelerated reaction at higher temperatures.

### 3.5. X-ray Diffraction of the Aluminum Orthophosphate Geopolymer Mortars

To analyze the conversion rate of aluminum orthophosphate through the geopolymerization reaction, the samples with a Si/Al ratio of 2/1 were assessed using the XRD method. This is possible because the geopolymer network is X-ray amorphous and the aluminum orthophosphate is crystalline. For metakaolin-based geopolymers, this will not work because metakaolin is already X-ray amorphous. The investigation took place after 7 days of hardening the geopolymer mortars for both curing methods, with the help of microwaves and at ambient temperature. At this point, only the proportions of unreacted aluminum orthophosphate and the amorphous proportions of the samples are considered.

As shown in Table 8, the proportion of X-ray amorphous phases is almost half the mass of the species in all the geopolymer mortars investigated. It is seen that the proportion of X-ray amorphous phases increases with the increasing alkalinity of the activator sodium silicate solution and, correspondingly, the amount of unreacted aluminum orthophosphate decreases. There is no significant difference in the phase components between the microwave and ambient temperature curing conditions, the proportions of the X-ray amorphous phase are almost identical.

### 3.6. Compressive Strength

**Figure 8:**
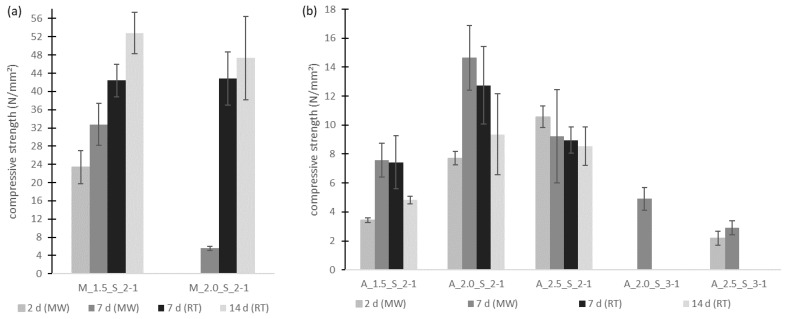
Compressive strengths of the geopolymer mortars with metakaolin (**a**) and aluminum orthophosphate (**b**).

The measured compressive strengths of the geopolymer mortars can be taken from Figure 8. If no results were noted, it was not possible to determine values because the samples were not yet well hardened. It could be assumed that the curing reaction would be slower for the samples cured in the laboratory environment at ambient temperatures, therefore, no strength test was performed on these after two days. For these samples, the compressive strength was additionally determined after 14 days.

As can be seen in Figure 8a, for metakaolin-based geopolymer mortars, the compressive strength obtained by curing at an ambient temperature was greater than that for samples cured using the microwave treatment. With the increasing age of the samples, the compressive strength increases. The greatest strength was achieved with the mixture M_1.5_S_2-1. After 7 days the microwave-treated samples reached a compressive strength of 32 MPa. The samples at the same age which had been stored at ambient temperature reached a compressive strength of 42 MPa. So, it can be assumed that through the faster evaporation of water the geopolymerization reaction changed. Water is not available long enough to be released stepwise by the passing condensation reaction, but evaporates too quickly. This has an impact on the geopolymer network, which cannot be built well and so the compressive strength decreases compared to that of the samples cured at ambient temperature.

For the microwave-treated metakaolin-based samples, after 7 days, compressive strengths of about 32 MPa (M_1.5_S_2-1) and about 5 MPa (M_2.0_S_2-1) were reached. In comparison, Chindaprasirt et al. reached a compressive strength after 7 days of approx. 9 MPa, 16 MPa and 17 MPa with microwave treatments of 90 W for 3 min, 5 min and 10 min. Their used geopolymers were prepared using coal fly ash, sodium hydroxide solution (10 M), sodium silicate solution (SiO_2_/Na_2_O mass ratio: 3.2) and graded river sand [29]. So, the obtained compressive strengths of Chindaprasirt et al. [29] lie between those of the samples tested in this study.

The results of the aluminum orthophosphate-based geopolymer mortars are shown in Figure 8b. For the Si/Al ratio of 2/1 for the aluminum orthophosphate-based geopolymer binders with microwave curing, a higher compressive strength was found compared to without the curing treatment. The greatest compressive strength was measured with a modulus of the sodium silicate solution of 2.0, with a value of 14.6 MPa after 7 days. Also, it can be seen that for samples cured at ambient temperatures the compressive strength decreases with increasing age. Compared to the Si/Al ratio of 3/1, the measured values with the ratio of 2/1 are higher. The curing of the samples with a ratio of 3/1 was not that fast, so there are no values for their curing at ambient temperatures. With the microwave treatment, the highest compressive strength of nearly 5 MPa was also reached with a sodium silicate solution modulus of 2.0 after 7 days (like for the Si/Al ratio of 2/1).

**Figure 9:**
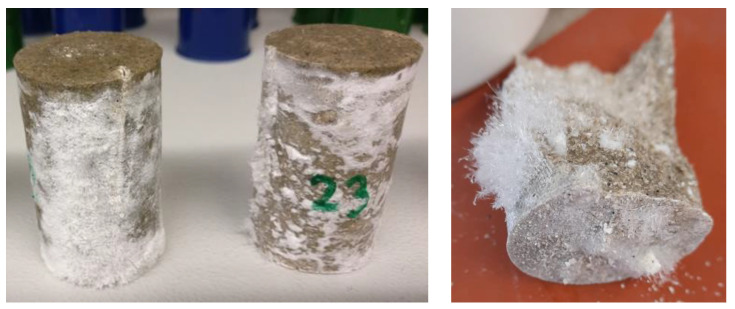
Salt efflorescence of the aluminum orthophosphate-based geopolymer binders.

In the aluminum orthophosphate-based geopolymer samples, agglomerates of unreacted aluminum orthophosphate were visible. Therefore, aluminum orthophosphate was not completely dissolved by the sodium silicate solutions and reacted incompletely. Some traits of an incomplete dissolution of aluminum orthophosphate in sodium silicate solution were also found by Partschefeld et al. [8]. Lagno et al. investigated the solubility of hydrated aluminum phosphate (AlPO_4_ × 1.5 H_2_O) at 22 °C at different pH values. The results showed that when the pH is over 4.0 the solution of hydrated aluminum phosphate becomes incongruent [43]. So, because the pH value of the geopolymer solutions is about 8 or higher (compare Table 9), the solution of the aluminum orthophosphate might be incomplete, leading to the formation of agglomerates. For the Si/Al value of 3/1 perhaps the lower content of aluminum orthophosphate cannot be dissolved completely due to agglomeration, and the small amount that is dissolved in the sodium silicate solution does not suffice to form a stabile geopolymer network. This was demonstrated by the salt efflorescence of the geopolymer samples (Figure 9), which showed after a few days, regardless of the sodium silicate solution that was used.

To determine what kind of salt efflorescence it was, crystals were stripped from the samples and an EDS analysis and an X-ray analysis were performed. The results showed that the efflorescence was composed of sodium carbonate and sodium phosphate hydrate. Sodium carbonate is formed by the reaction of a sodium compound from the geopolymer with carbon dioxide in the ambient air. Sodium phosphate hydrate is a reaction product of sodium ions from the sodium silicate solution with phosphate ions from aluminum orthophosphate. Through the formation of those salts, the ions are not fixed into the geopolymer network, resulting in cracks and lower strengths because of a lack of bonds.

In the comparison of both kinds of geopolymer binders it was found that the compressive strength of the metakaolin geopolymer mortar is generally higher than that of aluminum orthophosphate-based geopolymer mortars. One reason might be the agglomerates of unreacted aluminum orthophosphate in the aluminum orthophosphate-based geopolymer samples, as mentioned above. In contrast, for the metakaolin-based geopolymer mortars no agglomerates were visible. The metakaolin seems to be completely dispersed in the sodium silicate solutions, leading to a compact geopolymer network structure. The compact network structure of the metakaolin-based geopolymer binders is also reflected in the fact that there is no salt efflorescence here, but there is for the aluminum orthophosphate-based geopolymer mortars. So their geopolymer network is less stable. This could be another reason for the low strengths of the aluminum orthophosphate mortars.

### 3.7. Microstructure of the Geopolymers

To obtain more information about the microstructure of the hardened geopolymer mortars, they were viewed under SEM. The test was also performed with all samples that were hard enough for compressive strength testing.

Sun et al. reported that after the activator is added to the solid during geopolymer synthesis in the formed geopolymer gel some unreacted particles can be found. These particles can agglomerate on the geopolymer gel during the polycondensation process. Through microwave irradiation, the unreacted and aggregated particles can be polymerized and crosslinked, making the geopolymer network denser and more homogeneous and compact. Furthermore, the microwave treatment leads to the evaporation of water, which contributes to a denser microstructure by destroying pores [12].

**Figure 10:**
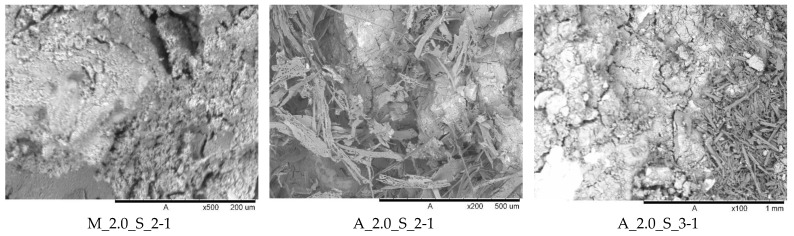
Microstructure of geopolymer mortars.

Because there were no obvious differences between the activators used, Figure 10 shows only the mortars activated with the sodium silicate solution with a modulus of 2.0. As can be seen for the metakaolin geopolymer mortars, the microstructure seems to be more homogeneous than for the aluminum orthophosphate-based mortars. Also, for both A_2.0_S samples needle-shaped salt efflorescence is recognizable. This topic was mentioned above. There is no significant visible difference between the two different Si/Al ratios. The different surface structures are the result of the different raw materials used.

The denser and more compact microstructure of the metakaolin-based geopolymer mortars corresponds to the higher compressive strengths of these mortars and the results of the ultrasonic transit time measurement. The inhomogeneous microstructure of the aluminum orthophosphate samples may be the result of salt efflorescence.

### 3.8. Stability Investigations Using Water Solubility Tests

Eluates were prepared from the cured geopolymer mortars used for the compressive strength test to test the leaching stability of those samples. The ions dissolved in the eluates were detected with the help of ICP-OES.

As shown in Table 9, all pH values are in the alkaline region. This is what was expected, because alkaline sodium silicate solutions were used as activators.

Moreover, Table 9 shows, for all samples, a very small amount of dissolved aluminum and silicon ions compared to the contained amounts. This shows that a very stable aluminosilicate geopolymer network was formed. In sample M_2.0_S_2-1, quite a high proportion of silicon ions were dissolved compared to the other geopolymer mortars investigated, which correlates with the low compressive strength of the sample and indicates a less stable network. The next highest proportion of silicon ions was dissolved in the aluminum orthophosphate-based geopolymer mortars with a Si/Al ratio of 3/1, which also showed low compressive strengths and, therefore, probably also exhibit a rather unstable geopolymer network. Likewise, the aluminum and silicon ions found could also originate from unreacted raw materials.

For the phosphate ions, only very low concentrations were measured in the eluates of the metakaolin-based geopolymer mortars, which correlates with the composition of the metakaolin. Very high levels of phosphate ions were dissolved out of the aluminum orthophosphate geopolymer mortars, almost the complete amount of phosphates they contained. The dissolved ions were also visible in the salt efflorescence already mentioned.

On the one hand, the sodium ions detected in the eluates may originate from crushed granite sand, which did not react in the geopolymerization and has a relatively high content of Na_2_O, as shown by its chemical analysis. On the other hand, the high amounts of sodium ions in the aluminum orthophosphate-based geopolymer mortars compared to the metakaolin-based geopolymer mortars, as well as the phosphate ions, might also result from dissolution due to the salt efflorescence.

## 4. Conclusions

This study dealt with the hardening and curing of geopolymer binders under the influence of elevated temperatures using a microwave oven. The results can be summarized as follows:

- With microwave treatment there is a fast rise in the sample temperatures, which accelerate the geopolymerization reaction.
- The kind of raw material and activator, the alkalinity of activator and the Si/Al ratio influence the curing and the properties of the geopolymer binders.
- Samples prepared with a sodium silicate solution with a modulus of 1.5 showed the highest compressive strengths.
- With aluminum orthophosphate, the curing and hardening of the geopolymers starts earlier than with metakaolin.
- Generally, the metakaolin-based samples showed a higher compressive strength, lower salt efflorescence and leachability, and a denser microstructure compared to samples based on aluminum orthophosphate.
- Aluminum orthophosphate-based geopolymer mortars with a Si/Al ratio of 3/1 exhibited very low compressive strengths compared to a Si/Al ratio of 2/1.

In summary, the microwave treatment of geopolymers achieves a very rapid solidification of the material. Within a few minutes the geopolymer is hardened and can be demolded. This is a great benefit for the manufacturing of prefabricated components and makes the geopolymers more accessible for the construction industry. Further demand on research exists in the influence of microwaves on the formed geopolymer structures, which could be conducted via ^29^Si and ^27^Al magnetic resonance spectroscopy. Due to their global availability, clay mixtures should also be investigated as base materials for geopolymer formation influenced by microwave radiation.

## Figures and Tables

**Table 1:** Chemical and mineralogical composition and properties of the used metakaolin.

Chemical composition (wt.%)		
SiO_2_		52.0
Al_2_O_3_		41.4
TiO_2_		0.93
Fe_2_O_3_		0.6
K_2_O		0.3
other oxides < 0.1 wt.%		0.33
drying loss		0.4
LOI (950 °C)		3.7
Mineralogical composition (wt.%)		
anatase	TiO_2_	0.9 ± 0.2
calcite	CaCO_3_	0.9 ± 0.4
kaolinite	Al_2_O_3_∙2SiO_2_∙2H_2_O	23.9 ± 1.5
quartz	SiO_2_	2.5 ± 0.3
amorphous		71.6 ± 1.5
Particle and surface properties		
BET surface ^1^ (m^2^/g)		11.5
particle size d_10_; d_50_; d_90_ (µm)		0.75; 5.35; 20.22
pure density ^2^ (g/cm^3^)		2.75

^1^ BET-measurement conditions: Nitrogen gas p_s_/p_0_ = 0.3, T_BET_ = 105 °C. ^2^ measured by Helium gas displacement.

**Table 2:** Chemical and mineralogical composition and properties of the used aluminum orthophosphate.

Chemical composition (wt.%)		
P_2_O_5_		57.0
Al_2_O_3_		35.9
SiO_2_		0.6
Na_2_O		0.15
other oxides < 0.1 wt.%		0.15
drying loss		0.9
LOI (950 °C)		12.2
Mineralogical composition (wt.%)		
berlinite (aluminum orthophosphate)	AlPO_4_	96.7 ± 1.2
aluminum metaphosphate	Al(PO_3_)_3_	3.3 ± 1.2
Particle and surface properties		
BET surface ^1^ (m^2^/g)		2.9
particle size d_10_; d_50_; d_90_ (µm)		1.73; 12.95; 94.17
pure density ^2^ (g/cm^3^)		2.56

^1^ BET-measurement conditions: Nitrogen gas p_s_/p_0_ = 0.3, T_BET_ = 105 °C. ^2^ measured by Helium gas displacement.

**Table 3:** Chemical and mineralogical composition and properties of the used crushed granite sand.

Chemical composition (wt.%)	
SiO_2_	67.3
Al_2_O_3_	14.8
K_2_O	4.27
Fe_2_O_3_	3.4
Na_2_O	3.29
CaO	2.4
MgO	1.4
P_2_O_5_	0.51
SO_3_	0.4
TiO_2_	0.29
MnO	0.18
other oxides < 0.1 wt.%	0.02
drying loss	0.1
LOI (950 °C)	1.6
Mineralogical composition (wt.%)	
quartz	27.7 ± 1.0
orthoclase	21.6 ± 1.5
plagioclase albite	28.4 ± 2.4
plagioclase andesine	6.7 ± 2.3
muscovite	11.8 ± 1.5
chlorite	3.5 ± 10.9
pyrite	0.3 ± 0.1
Surface properties	
BET surface ^1^ (m^2^/g)	2.5

^1^ Nitrogen gas p_s_/p_0_ = 0.3, T_BET_ = 105 °C.

**Table 4:** Total microwave irradiation times of samples (s).

	WGM = 1.5	WGM = 2.0	WGM = 2.5
M_WG_S_2-1	360	810	--- ^1^
A_WG_S_2-1	162	270	306
A_WG_S_3-1	--- ^1^	576	720

^1^ The samples were not hardened after 27 min total irradiation time, so the experiment was terminated for these samples.

**Table 5:** Dielectric properties of selected geopolymer pastes.

Material	Frequency (MHz)	Dielectric Constant	Dielectric Loss Factor
M_1.5_2-1 (fresh)	915	24.51	1.17
	2450	18.29	0.95
M_1.5_2-1 (after 1 h)	915	11.96	0.73
	2450	9.45	0.68
A_2.0_2-1 (fresh)	915	23.44	1.13
	2450	16.75	0.97
A_2.0_2-1 (after 1 h)	915	17.94	0.68
	2450	13.59	0.66

**Table 6:** Temperature development of the geopolymer pastes.

Material	Start Temperature (°C)	End Temperature (°C)	Total Microwave Irradiation Time until Hardening (s)
M_1.5_2-1	24.0	70.3	65
M_2.0_2-1	24.0	96.5	--- ^1^
M_2.5_2-1	24.3	95.1	--- ^1^
A_1.5_2-1	35.5	60.3	55
A_2.0_2-1	30.9	69.7	60
A_2.5_2-1	27.1	70.3	70
A_1.5_3-1	31.2	78.4	--- ^1^
A_2.0_3-1	28.3	80.6	--- ^1^
A_2.5_3-1	26.7	78.7	--- ^1^

^1^ no hardening after 120 s of irradiation time (end of measurement).

**Table 7:** Start of the formation of the geopolymer network.

	Onset Point (min)		
	40 °C	60 °C	80 °C
M_1.5_2-1	46	9	3
M_2.0_2-1	59	29	3
A_1.5_2-1	2	1	---
A_2.0_2-1	14	5	1
A_2.5_2-1	9	4	2
A_1.5_3-1	31	16	5
A_2.0_3-1	40	9	3
A_2.5_3-1	21	12	3

**Table 8:** Phase compositions of the aluminum orthophosphate geopolymer mortars (Si/Al = 2/1).

Sample	Phase Composition (wt.%)	
	X-ray Amorphous Phases	Residual AlPO_4_ (Berlinite)
A_1.5_S_2-1	53.9 ± 13.5	1.6 ± 0.4
A_2.0_S_2-1	48.0 ± 1.9	4.6 ± 0.6
A_2.5_S_2-1	43.8 ± 2.0	3.7 ± 0.6
A_1.5_S_2-1_RT	53.5 ± 1.7	2.0 ± 0.4
A_2.0_S_2-1_RT	51.0 ± 1.8	4.3 ± 0.6
A_2.5_S_2-1_RT	43.7 ± 2.1	7.7 ± 0.9

**Table 9:** Proportions of ions in the eluates and oxides in the geopolymer mortars.

Sample	M_1.5_S_2-1	M_2.0_S_2-1	A_1.5_S_2-1	A_2.0_S_2-1	A_2.5_S_2-1	A_2.0_S_3-1	A_2.5_S_3-1
oxides/compounds in the geopolymer mortars (wt.%)							
Al_2_O_3_	15.14	15.37	10.04	10.47	10.95	8.45	8.92
SiO_2_	51.78	52.96	37.16	39.04	40.48	35.04	38.22
P_2_O_5_	0.29	0.29	7.32	7.63	7.98	6.16	6.50
Na_2_O	1.75	1.77	1.25	1.31	1.37	1.05	1.11
NaOH	6.18	4.99	8.42	8.42	6.86	12.16	8.39
chemical species in the eluates (wt.% related to contained quantities)							
Al	0.03	0.003	0.05	0.03	0.07	0.002	0.002
Si	0.12	1.23	0.02	0.04	0.04	0.66	0.81
PO_4_	0.02	0.02	10.16	9.15	7.67	9.43	6.60
Na	1.36	1.87	4.73	3.88	3.02	4.96	3.56
pH	10.8	11.2	9.6	8.8	8.4	10.4	10.3

## Data Availability

Data are contained within the article.
